# Hospital Meetings, &c.

**Published:** 1900-06-23

**Authors:** 


### HOSPITAL MEETINGS, &C.

### THE GROSVENOR HOSPITAL FOR CHILDREN.

THE annual general meeting of the governors of the hospital was held on Wednesday, June 13th, 1900, Viscount MIDLETON in the chair. Viscount Midleton moved that the report in the hands of those present be taken as read and adopted. He said that in a hospital of this description there were two things to consider. Firstly, Was there any real need for the hospital in this neighbourhood? and, secondly, Were the means used in the working of the hospital the best possible? To both of these questions he thought he could truthfully answer, "Yes." In the first place, 189 in-patients were treated in the hospital during the year 1899, while the number of out-patients' attendances ran into thousands. With regard to the in-patients, he might say that they were drawn from a class of people who could not afford to pay a doctor's fee, and could not possibly be treated in their own homes owing to lack of accommodation. He reminded the meeting that there were 30 beds in the hospital, nine of which remained vacant through lack of funds, and said that, having perused the report very carefully, he was convinced that at least £900 per annum increase in subscriptions was required if the hospital was to do the work for which it was intended. He appealed to those present to induce their friends to subscribe to this most worthy institution. Sir Charles Fremantle seconded the motion, which was carried. On the motion of Sir Charles Fremantle, a proposal that, "Owing to a difficulty in obtaining the higher number that No. 17 Rule should now read: 'That the institution shall be under the management of a general committee, which shall consist of not less than seven governors,'" was carried. Mr. Arbuthnot proposed a vote of thanks to Viscount Midleton for his kindness in coming down to preside at the meeting. He pleaded for new annual subscriptions, and mentioned Lady Aberdeen's generous offer of raising the £300 that she had already given to the endowment fund to £1,000, provided the hospital could raise £10,000 by May, 1902. This, he said, ought to be done, as if the hospital had a regular income it would to a very great extent end the anxiety as to its financial condition, which he, as the treasurer, felt. On Sir W. F. Clarke seconding the motion, which was carried, the proceedings terminated.

